# An Analysis of BMP1 Associated with m6A Modification and Immune Infiltration in Pancancer

**DOI:** 10.1155/2022/7899961

**Published:** 2022-10-11

**Authors:** Yiyi Zhou, Shuangshuang Xu, Qinying Sun, Yuchao Dong

**Affiliations:** Department of Respiratory and Critical Care Medicine, Changhai Hospital, Shanghai, China

## Abstract

**Background:**

This research explores the underlying link between diagnosis and therapy between bone morphogenetic protein 1 (BMP1) and various cancers.

**Methods:**

Three immunotherapeutic cohorts, by the composition of IMvigor210, GSE35640, and GSE78220 were obtained from previously published articles and the Gene Expression Omnibus database. The different expressions of BMP1 in various clinical parameters were conducted, and prognostic analysis was executed utilizing Cox proportional hazard regression and Gene Expression Profiling Interactive Analysis. Moreover, the correlation between BMP1 and tumor microenvironment was analyzed using ESTIMATE and CIBERSORT algorithms. Tumor mutational burden and microsatellite instability were also included. The correlation between m6A modification and the gene expression level was analyzed using Tumor IMmune Estimation Resource, the University of Alabama at Birmingham Cancer data analysis portal. Gene Set Cancer Analysis analyzed the correlation of BMP1 expression level with copy number variations and methylation. Furthermore, the correlation between BMP1 and therapeutic response after antineoplastic drug use was illustrated for further discussion.

**Results:**

BMP1 expression had significant differences in 14 cancers. It presented an intimate relationship with immune-relevant biomarkers. A variation analysis indicated that BMP1 had a significant association with immunotherapeutic response. The expression level of BMP1 was closely associated with insulin-like growth factor binding protein 3, an m6A modification relative gene. Except for a few cancer types, methylation negatively correlated with BMP1, and copy number variations positively correlated with BMP1. Notably, low BMP1 expression was connected with immunotherapeutic response in the cohorts, and its expression was related to increased sectional sensitivity of drugs.

**Conclusion:**

BMP1 may serve as a potential biomarker for prognostic prediction and immunologic infiltration in diversified cancers, providing a new thought approach for oncotherapy.

## 1. Introduction

Bone morphogenetic proteins (BMPs), which have been isolated and cloned from the extracellular matrix (ECM), are bone-inducing molecules [1]. The transforming growth factor-beta (TGF-*β*) superfamily contains BMPs family, ranging from BMP2 to BMP16 [2]. Unlike the other bone morphogens, the bone morphogenetic protein 1 (BMP1) is not just like TGF-*β* but belongs to a putative proteases family involved in pattern formation during development in diverse organisms [3]. A previous study indicated that BMP1 mediates the cleavage of carboxyl-terminal (C-term) propeptides from procollagens is a critical process in fibrillar collagen fiber formation [4], and it appears to be the primary enzyme responsible for the fibrillar type (I, II, III, V, and VII) procollagen maturation [5, 6]. Moreover, BMP1 has been implicated in tumor progression by inducing the initial cleavage and release of complexes [7]. It has been proved in some cancer types utilizing multiple pieces of research. For example, LOX/BMP1 mediates the malignant progression of anaplastic thyroid carcinoma [8], and BMP1 is considered a promoting factor in breast cancer and bone metastasis [9, 10]. A high BMP1 expression reflects a poor prognosis in human gastric cancer and clear cell renal cell carcinoma [2, 11]. Furthermore, increased BMP1 expression and activity are associated with the malignant grade of astrocytomas, which may be related to therapy [12]. On the contrary, some previous studies implicated that BMPs induce apoptosis and inhibit the proliferation of tumors, such as increasing the expression of BMP1 can inhibit the migration of tumors by restraining the process of EMT in colorectal cancer [13, 14]. A recent study implicated the upregulation of EMT transcription factors (BMP1, CDH2, KRT14, etc.) to high proliferation rates, mechanical molecular barriers disassembly, and increased cancer cell motility, leading to metastasis risk in pulmonary neuroendocrine neoplasms [15]. BMP1 is a potential biomarker and mediator in diversified malignant tumors, whereas the function and pathway of its impact on pancancer remain unclear.

In the 1960s, scientists discovered modified nucleotides in many cellular RNAs [16], such as N1-methyladenosine (m1A), 5-methylcytidine (m5C), and N6-methyladenosine (m6A) [17–20]. RNA-modifying proteins (RMPs) involved in the process of RNA methylation are called “writers,” “readers,” and “erasers.” [21]. Currently, m6A is not only the most abundantly modified mRNA but also the best characterized at the functional level [22]. Since the discovery of the first gene in m6A modification, METTL3, other genes have been reported one after another, including m6A “writers” (METTL14, METTL16, WTAP, VIRMA, ZC3H13, CBLL1, RBM15, and RBM15B), m6A “readers” (YTHDC1-2, YTHDF1-3, HNRNPC, FMR1, LRPPRC, HNRNPA2B1, IGFBP1-3, RBMX, ELAVL1, and IGF2BP1), and m6A “erasers” (FTO and ALKBH5) [23–28]. Nevertheless, the relationship between m6A regulators and BMP1 expression in pancancer is unclear.

The BMP1 expression landscape of 33 cancer types was presented in our study. First, we explored the correlation of BMP1 expression with the prognosis of multiple cancers and N6-methyladenosine-related factors. Whereafter, the latent influences of BMP1 were analyzed, respectively, acting on tumor microenvironment, TMB, and MSI. More interestingly, the expression of BMP1 was prominently related to tumor immune checkpoint blockade treatment. In addition, the association between BMP1 expression and drug sensitivity was investigated. Overall, the findings uncovered new evidence of the significant part of BMP1 in pancancer.

## 2. Methodology

### 2.1. Dataset Acquisition

The gene expression data and clinicopathological information of 33 cancers were obtained from the University of California Santa Cruz Xena Explorer (UCSC), Genotype-Tissue Expression (GTEx) (https://www.gtexportal.org/) database, and the Cancer Genome Atlas (TCGA) (https://portal.gdc.cancer.gov/) database. The format of the downloaded data was log_2_TPM, which was standardized by R software. In addition, three immunotherapeutic cohorts (GSE35640, GSE78220, and IMvigor210) were identified by a systematic search in the Gene Expression Omnibus database (GEO) and previously published research [29, 30]. Microarray data for GSE35640, including 34 nonresponders and 22 responders, focused on MAGE-A3 immunotherapy in metastatic melanoma, while GSE78220 contained 13 nonresponders and 15 responders explored MAPK-targeted therapy in melanoma. The IMvigor210 cohort containing 230 nonresponders and 68 responders was about urothelial cancer with atezolizumab intervention.

### 2.2. Investigation of BMP1 Expression and Activity in Pancancer

The “limma” R package and GEPIA, based on TCGA and GTEx data, were used to investigate BMP1 expression levels in normal and tumor tissues. The subgroup analysis of multiple clinic-pathological features, including age, cancer stages, and gender, were further conducted. Using the “survival” package, researchers investigated the prognostic prediction of BMP1 in 33 types of cancer using univariate Cox regression analysis. Single sample gene set enrichment analysis (ssGSEA) was implemented to detect the divergence of the BMP1 activity in normal and tumor samples. Subsequently, the mean value of the gene expression and activity was sorted and calculated in pancancer to explore potential features of BMP1.

### 2.3. Correlation Analysis with Immune-Related and N6-Methyladenosine-Related Factors

The association between BMP1 expression and 20 m6A relative genes (ALKBH5, ELAVL1, FMR1, FTO, HNRNPA2B1, HNRNPC, IGF2BP1, IGFBP1/2/3, METTL3/14, RBM15/15B, WTAP, YTHDC1/2, and YTHDF1/2/3) was analyzed utilizing the TIMER2 tool (http://http://timer.cistrome.org/) [31]. The Kaplan–Meier curve depicted the association between the survival probability of patients in pancancer and the expression level of m6A-related factors. UALCAN (http://ualcan.path.uab.edu/index.html) was utilized for visual analysis of the cohort. Infiltrating stromal and immune cells forming the major fraction of normal cells in cancers have been proved to perturb the tumor signal and play a significant role in oncobiology [32]. Estimating stromal and immune cells in malignant tumors using the expression data (ESTIMATE) algorithm effectively determines the fraction of stromal and immune cells in tumor tissues [33]. CIBERSORT is a deconvolution algorithm for characterizing the cell composition of complex tissues from the gene expression profiles [34]. The enumeration of stromal and immune scores was applied using “ESTIMATE” R package to predict the level of infiltrating stromal and immune cells. The complex associations between BMP1 expression and 22 distinct leukocyte subsets were identified by applying the CIBERSORT algorithm. TISIDB (http://cis.hku.hk/TISIDB/) integrating multiple heterogeneous data types is an integrated repository portal for tumor and immune system interaction [35]. Later, this online database used heatmaps and scatter plots to present the underlying correlation between BMP1 expression and immunological modulators. MSI and TMB, two immune-relevant biomarkers for checkpoint blockade immunotherapy response, have been linked to clinical response to immunotherapy [36]. Thus, the study explored the pertinence between BMP1 expression and the two immune-relevant biomarkers using R software. According to the exome size, the mutation counts were divided to calculate TMB of 33 cancers. The MSI score obtained from previously published research [37] and BMP1 expression were also analyzed using R software. At last, GSEA investigated the relevant signaling pathways between the high BMP1-expressing and low BMP1-expressing clusters. The five highest enrichment scores of pathways were considered.

### 2.4. Immunotherapeutic Response and Drug Sensitivity Analysis

For cancer therapies, tumor shrinkage categorized as partial response (PR) or complete response (CR) is considered an essential evaluation [38]. Patients who achieved CR or PR in this study were categorized as responders. The rest were listed as nonresponders. Three relevant immunotherapeutic cohorts mentioned before were applied to distinguish discrepancies in the expression level of BMP1 between the responder and nonresponder clusters using the Wilcoxon test. In addition, drug sensitivity data were available from the CellMiner dataset. Excluding those failing to pass FDA-approved and clinical trials, the research probed into the association between drug sensitivity and BMP1 expression utilizing R software.

### 2.5. BMP1 CNV and Methylation Profile Analysis

Based on the TCGA database, the Gene Set Cancer Analysis (GSCA) [39] platform offers copy number variation (CNV) and methylation to explore and analyze the expression of BMP1 ulteriorly. The connections of BMP1 mRNA expression with BMP1 CNV and methylation in diversified cancer types were investigated thoroughly utilizing GSCA data.

## 3. Results

### 3.1. Clinical Landscape of BMP1 Expression in Pancancer

**Figure 1:**
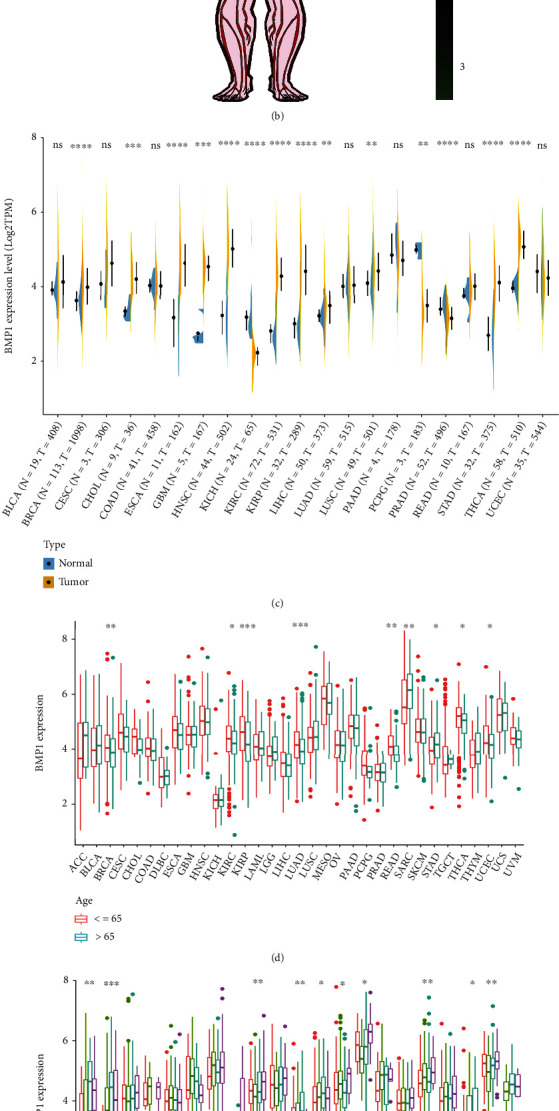
Association between expression levels of BMP1 and clinical modules in various cancers. (a, b) The anatomy diagram of male and female expression, respectively. (c) The expression levels of BMP1 in tumor vs. normal tissues. (d) The comparation of BMP1 expression between two age groups, demarcation line was formed by age 65. (e) The correlation between BMP1 and neoplasm staging. (f) The comparation of BMP1 expression between male group and female group. ^∗^*p* < 0.05, ^∗∗^*p* < 0.01, and ^∗∗∗^*p* < 0.001.

The corresponding full names of pancancer are summarized and presented in this article (Table 1) [29]. We obtained BMP1 expression in various human tissues to understand the key factors in signaling pathways ulteriorly (Figures 1(a) and 1(b)). The anatomy diagram of males showed low BMP1 expression in the brain, pancreas, and muscle, while high expression in adipose tissue, nerve, and prostate. The anatomy diagram of females showed low BMP1 expression in the brain, pancreas, and muscle, while high expression in the uterus, cervix, vagina, nerve, and fallopian tube. We discovered that the BMP1 expression level in the generative organ was higher in females than in males. BMP1 was notably overexpressed in 11 carcinomas presented by the violin plot, with high expression levels in BRCA, CHOL, ESCA, GBM, HNSC, KIRC, KIRP, LIHC, LUSC, STAD, and THCA, and low expression in 3 cancer types, including KICH, PCPG, and PRAD (Figure 1(c)). As presented in Figure 1(d), BMP1 was differentially overexpressed among younger patients (age ≤ 65) of the BRCA, KIRC, KIRP, LUAD, READ, THCA, and UCEC cases; whereas, it was significantly overexpressed in STAD and SARC groups (age > 65). In addition, the subgroup analysis in neoplasm staging showed the gene expression had significant correlations with ACC, BLCA, KIRC, LIHC, LUAD, LUSC, MESO, SKCM, TGCT, and THCA (Figure 1(e)). Besides, the BMP1 expression differed significantly with COAD, LIHC, and SARC based on gender cohorts (Figure 1(f)).

**Figure 2:**
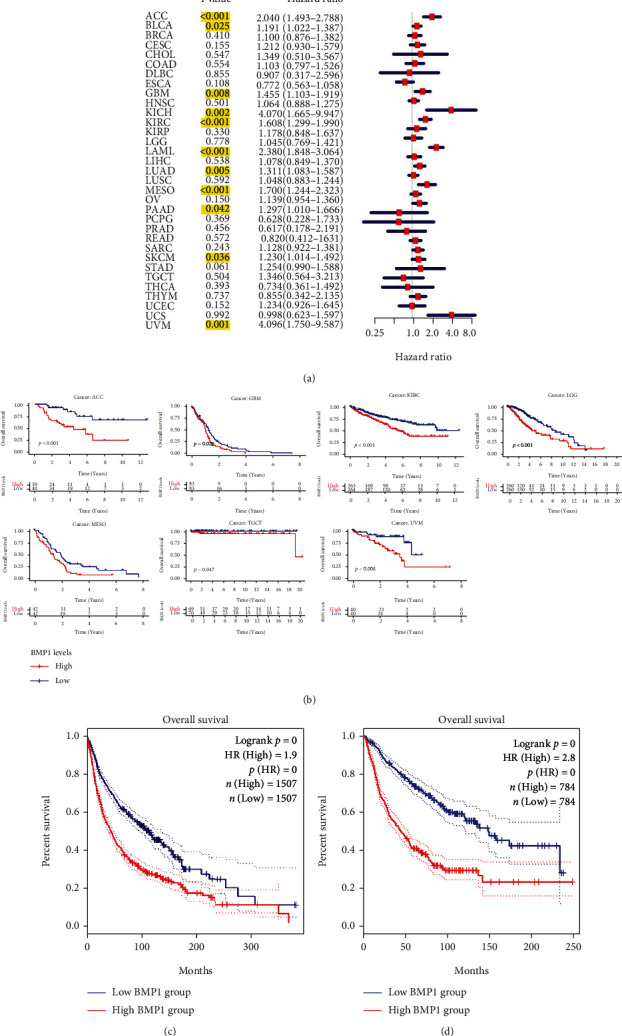
The correlation between BMP1 and overall survival (OS). (a) The forest plot was conducted using “forestplot” package. The yellow marks meant the cancers with significant association between BMP1 and prognostic indicators. Hazard ratio (HR) greater than 1 considered the BMP1 expression as a contributed element to demise. (b) The survival curves were pictured utilizing Kaplan–Meier survival analysis. (c) The OS of 11 cancer types based on the forest plot using GEPIA data, and (d) 7 cancers based on K–M plot using GEPIA data, considering *p* < 0.05 as significant difference.

Subsequently, forest plots and Kaplan–Meier plots were drawn using the “forestplot” and “survival” packages to present the relationship between BMP1 and prognostic indicators, consisting of overall survival (OS), progression-free survival (PFS), and disease-free survival (DFS). A positive correlation between the expression level of BMP1 and OS emerges in ACC, BLCA, GBM, KICH, KIRC, LGG, LUAD, MESO, PAAD, SKCM, and UVM (Figure 2(a)). The Kaplan–Meier plots in Figure 2(b) indicate that seven kinds of tumors (ACC, GBM, LGG, KIRC, MESO, TGCT, and UVM) with high BMP1 expression had poor OS. Two graphs showed the OS after multiple neoplasms based on Figures 2(a) and 2(b) (Figures 2(c) and 2(d)).

**Figure 3:**
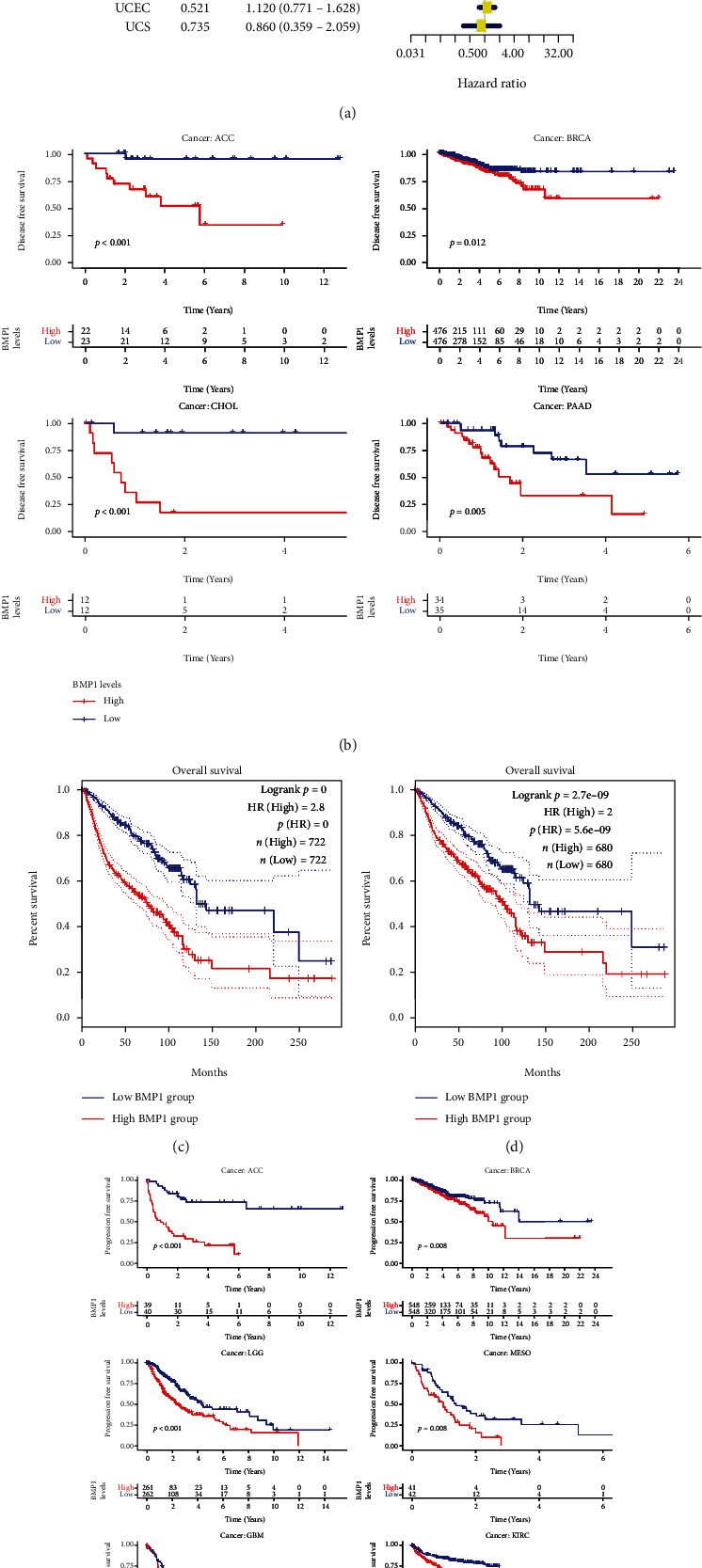
The correlation between BMP1 and disease-free survival (DFS). (a) The forest plot was conducted using “forestplot” package. The yellow marks meant the cancers with significant association between BMP1 and prognostic indicators. (b) The survival plots were presented based on the Kaplan–Meier survival methods. (c) The DFS of 5 cancers based on the forest plot using GEPIA data, and (d) 4 types of cancers based on K–M plot using GEPIA data. (e) The progression-free survival (PFS) of pancancer was utilized by Kaplan–Meier survival methods based on R package, considering *p* value <0.05 as significant difference.

Besides, a positive association between BMP1 and DFS was presented in ACC, BRCA, CHOL, MESO, and PAAD (Figure 3(a)). Furthermore, these cancers had an inverse effect on BMP1 expression (Figure 3(c)). Based on K–M plots, a positive association between BMP1 and DFS was presented in ACC, BRCA, CHOL, and PAAD (Figure 3(b)). Likewise, BMP1 expression had inversely affected these cancers (Figure 3(d)). In contrast, the PFS K–M plots confirmed that high expression levels significantly affected PFS in ACC, BRCA, GBM, KIRC, LGG, MESO, PAAD, and TGCT (Figure 3(e)). Combined with the above results, our findings indicated that patients with BMP1 overexpression got a poor prognosis in various carcinoma, including ACC, BRCA, GBM, KIRC, LUAD, PAAD, and MESO.

**Figure 4:**
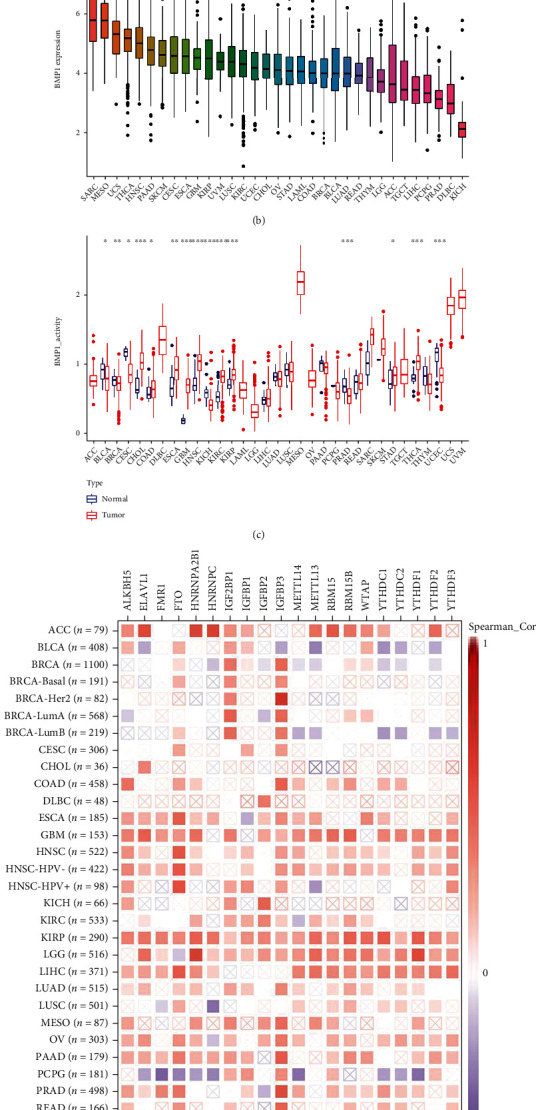
The investigation of gene activity (a–c) and correlation between BMP1 expression and m6A-related factors (d). (a) Mean activity in 33 types of carcinomas. (b) Mean expression in 33 types of carcinomas. (c) Differential activity analysis in tumor groups versus normal groups. (d) The correlation between the gene and m6A markers based on TIMER2 platform (^∗^*p* < 0.05; ^∗∗^*p* < 0.01; and ^∗∗∗^*p* < 0.001).

The genes most related to BMP1 were found using coexpression analysis, and these genes were used as active genes of BMP1 to obtain gene set files of active genes. Then, gene set files were analyzed by ssGSEA to obtain BMP1 activity scores for each sample. The box plot results showed that BMP1 activity and expression were higher in MESO, UCS, and SARC (Figures 4(a) and 4(b)). Moreover, the activity of BMP1 was notably decreased in the cancer groups of BLCA, BRCA, CESC, KICH, PRAD, and UCEC. Meanwhile, it increased in the CHOL, COAD, ESCA, GBM, HNSC, KIRC, KIRP, STAD, and THCA tumor groups (Figure 4(c)).

### 3.2. The Association of BMP1 Expression Level with N6-Methyladenosine-Related Markers and Immune-Related Factors

**Figure 5:**
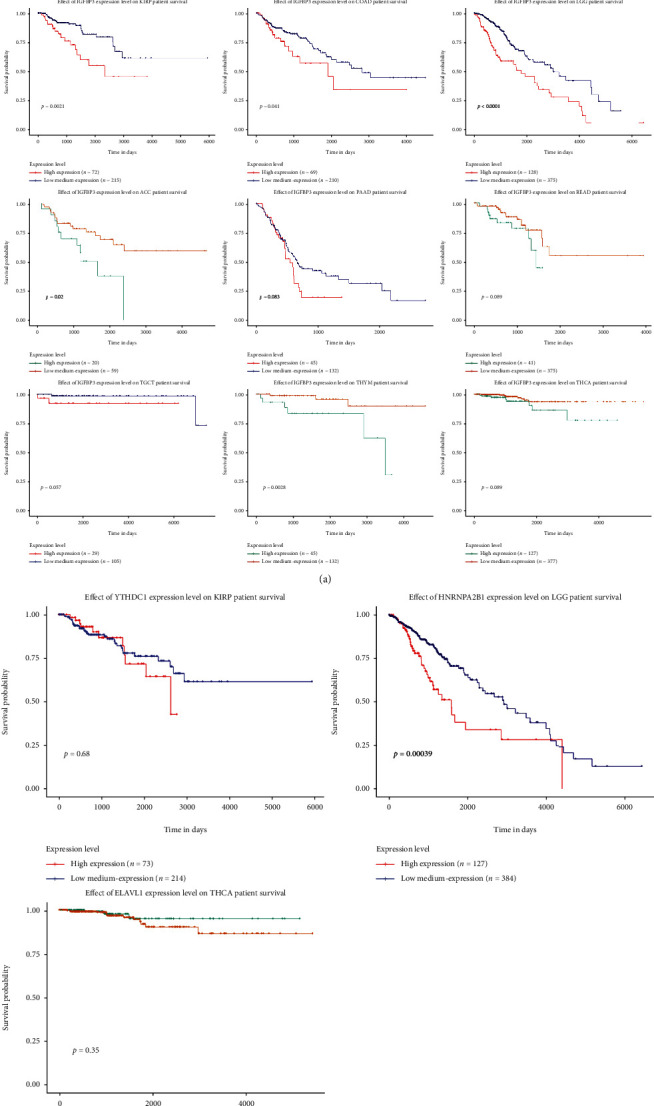
The relationship between BMP1 expression and m6A-related markers in pancancer. (a, b) The survival curves of IGFBP3, YTHDC1, HNRNPA2B1, and ELAVL1; the results were statistically significant (*p* < 0.05).

The m6A modification has a substantial impact on the progression of pancancer. Hence, the correlation between BMP1 expression and 20 types of m6A-related factors was explored systematically. As illustrated in Figure 4(d), the expression level of BMP1 had a significantly positive correlation with IGFBP3. Furthermore, BMP1 expression was positively related to all the 20 m6A relative markers in KIRP. There into, it had the most significant correlation with YTHDC1. In LGG and THCA, most m6A relative markers were also positively associated with BMP1. HNRNPA2B1 and ELAVL1 were the most significant genes. The Kaplan–Meier curves stated that the expression of IGFBP3 was remarkably associated with poor prognosis of various tumors (Figure 5(a)), including ACC, COAD, KIRP, LGG, MESO, THYM, and THCA. Moreover, the positive relationship between the high expression of HNRNPA2B1 and poor prognosis in LGG is revealed in Figure 5(b). Nevertheless, the relationships between YTHDC1, poor prognosis in KIRP and ELAVL1, and poor prognosis in THCA were insubstantial. It pointed out that there may be an underlying correlation between the m6A modification and BMP1 expression in various cancers, particularly impacting the pullulation and prognosis of those cancers through regulating IGFBP3.

**Figure 6:**
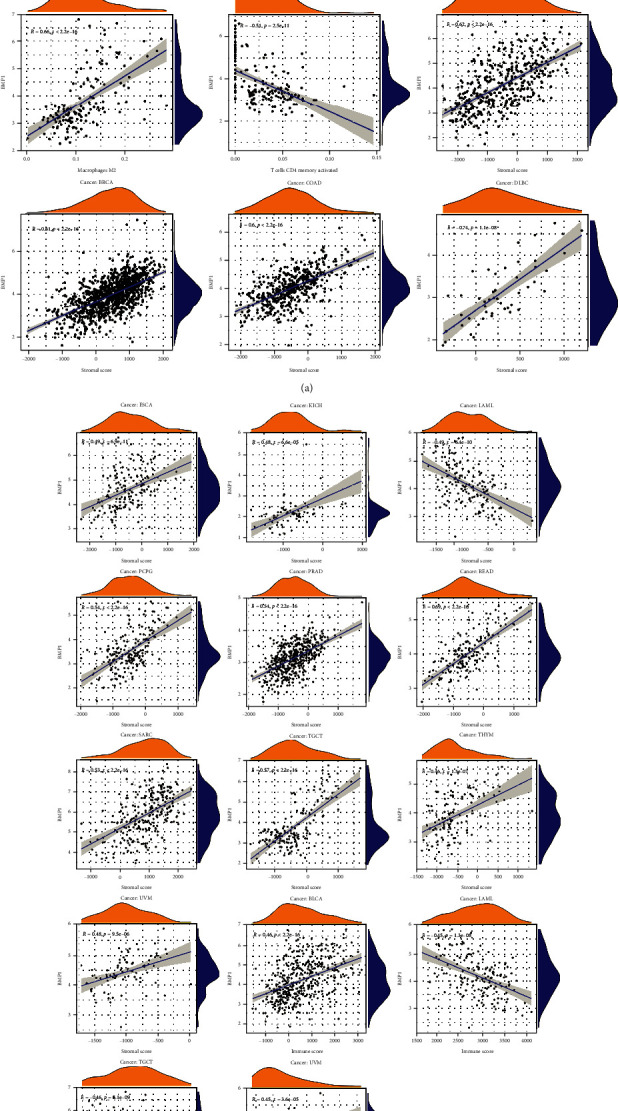
The expression of BMP1 associated with immune cell infiltration and ESTIMATE score. (a, b) The numeration of immune cell infiltration was utilized by CIBERSORT algorithm, and scatter plots were structured with the limiting condition (R > 0.5 and *p* < 0.001). The ESTIMATE score consisted of tumor purity, stromal score, and immune score. The correlation scatter plots were illustrated with the limiting condition (R > 0.44 and *p* < 0.001).

The immune cell infiltration landscape was investigated to discover the correlation between the immune cells and gene expression in 33 cancer groups using the CIBERSORT algorithm. The scatter plots demonstrated a positive relationship between BMP1 and M0 macrophage content in DLBC and a negative correlation with resting memory CD4 T cells in ACC. In TGCT, BMP1 was positively associated with M2 macrophage infiltration and negatively associated with activated memory CD4 T cell and naive B cell infiltration (Figure 6(a)). Afterward, the correlations between BMP1 expression, stromal score, and immune score were established in Figures 6(a) and 6(b). As the scatter plots presented, the expression level of BMP1 had significant correlations with 14 cancer stromal scores (BLCA, BRCA, COAD, DLBC, ESCA, LAML, KICH, PCPG, PRAD, READ, SARC, TGCT, THYM, and UVM). In addition, it was significantly related to the BLCA, LAML, TGCT, and UVM immune scores.

**Figure 7:**
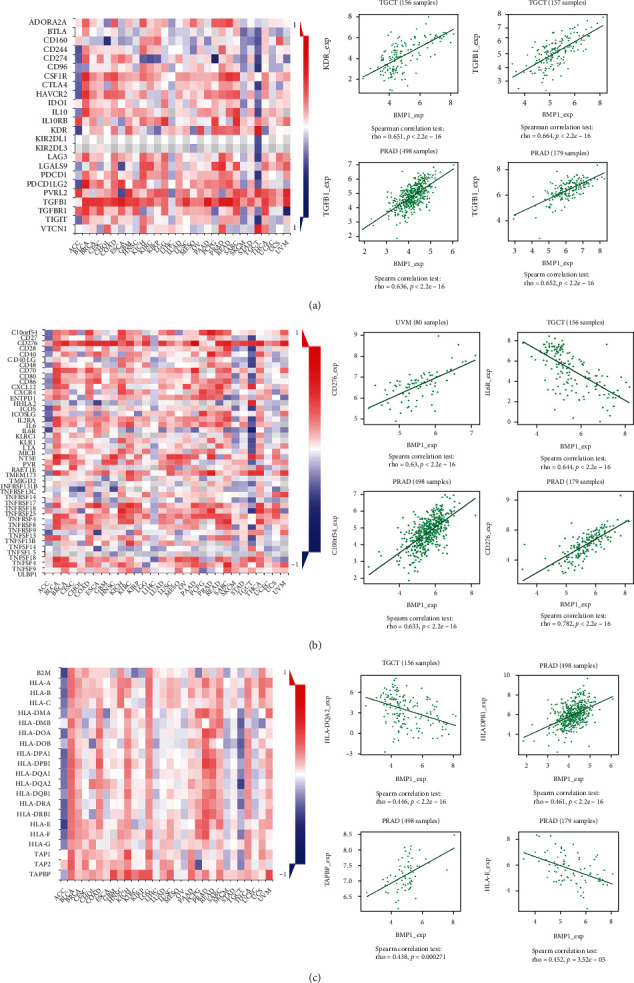
Exploration about the relationship between BMP1 and immune inhibitors, immune stimulators, and MHC molecules (a–c). The vermilion represented positive correlations, whereas blue meant negative correlations. The 4 dot plots on the right represented the top 4 significant associations.

**Figure 8:**
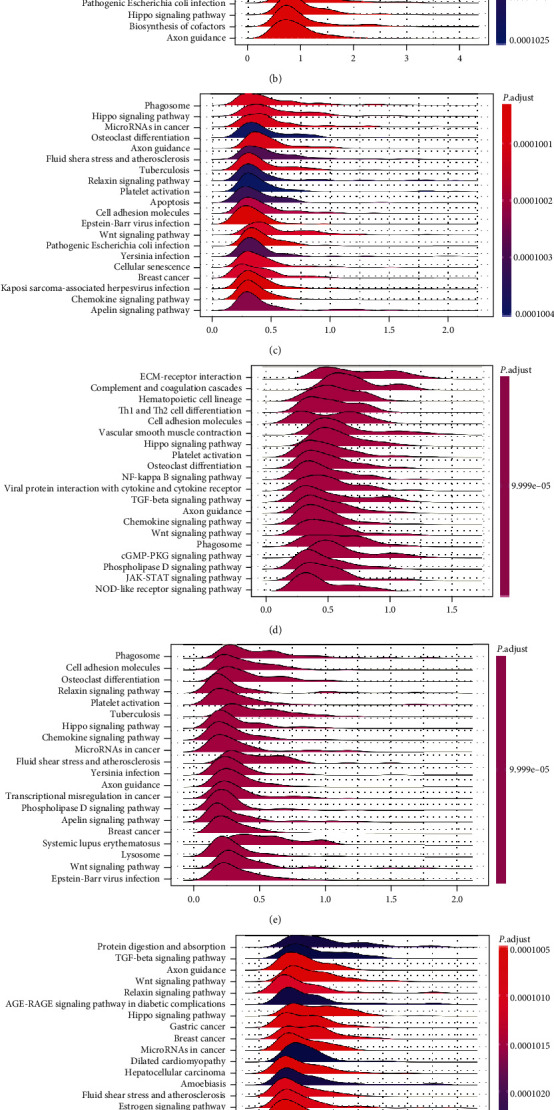
The Kyoto Encyclopedia of Genes and Genomes (KEGG) pathway enrichment analysis based on GSEA. Each ridgeline plot represented KEGG pathways in corresponding cancer, plotted by enrichment score on the horizontal axis and KEGG pathways on the vertical. (a)ACC; (b)KICH; (c)PAAD; (d)PRAD; (e)READ; (f)TGCT; and (g)UVM.

**Figure 9:**
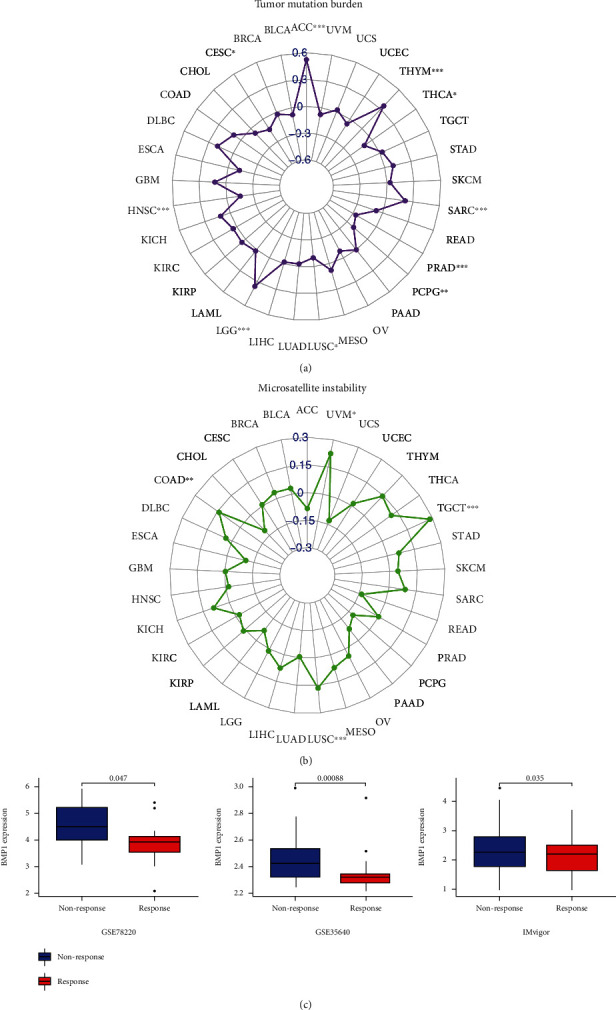
The analysis of BMP1with two immunotherapeutic markers (TMB and MSI) (a, b) and the immunotherapeutic response (c). These three cohorts were all divided into the response and the nonresponse groups. GSE78220 explored for MAPK-targeted therapy in melanoma. GSE35640 focused on MAGE-A3 immunotherapy in metastatic melanoma. IMvigor210 was about urothelial cancer with atezolizumab intervention. ^∗^*p* < 0.05, ^∗∗^*p* < 0.01, and ^∗∗∗^*p* < 0.001.

We investigated the association between the gene expression level and immune modulators based on the TISIDB database.  Figure 7(a) displays that BMP1 expression was positively related to TGFB1 in PAAD, PRAD, and READ, as well as KDR in TGCT, in analyses of 24 immune inhibitors. The heatmap in Figure 7(b) illustrated that BMP1 expression had significant positive correlations with CD276 in PAAD and UVM and C10orf54 in PRAD. In contrast, a negative association between BMP1 and IL6R in TGCT is also illustrated. Like the previous two heatmaps, the expression of BMP1 had notably positive associations with TAPBP in KICH and HLA-DPB1 in PRAD. Meanwhile, BMP1 expression negatively correlated with HLA-E in ACC and HLA-DQA2 in TGCT (Figure 7(c)). The above conclusions suggested robust correlations between BMP1 and ACC, KICH, PRAD, READ, PAAD, TGCT, and UVM. Thus, we conducted a GSEA analysis to probe the underlying pathways involved in BMP1 signaling in those cancers mentioned before. The analysis according to ridgeline plots in Figure 8(a)–8(g) demonstrates that the gene was mainly involved in immune-related pathways. For instance, inducing various cancers and inflammation, hippo-signaling pathways were enriched in ACC, KICH, PRAD, READ, PAAD, and TGCT, separately. Furthermore, the expression was closely related to the two dynamic biomarkers of the immune checkpoint blockade. The radar maps in Figure 9(a) indicate positive correlations between BMP1 and TMB in ACC, LGG, SARC, and THYM, whereas a negative correlation can be noticed in CESC, HNSC, LUSC, PCPG, PRAD, and THCA. A positive correlation was observed between MSI and BMP1 in COAD, LUSC, TGCT, THCA, and UVM (Figure 9(b)).

### 3.3. Immunotherapeutic Response and Drug Sensitivity

**Figure 10:**
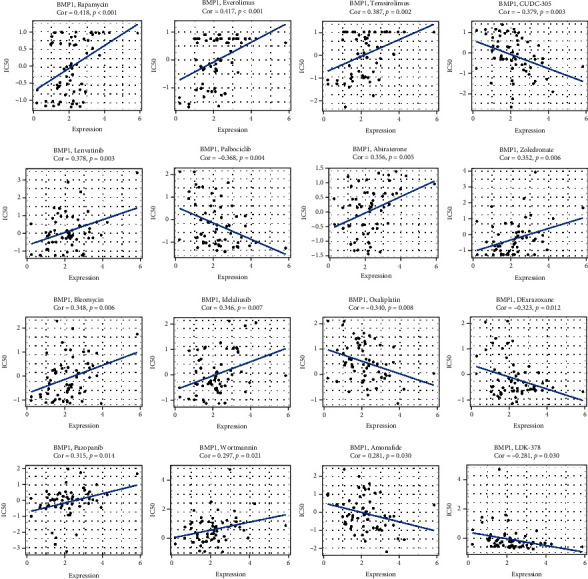
The correlation between drug sensitivity and BMP1. The expression levels were significantly associated with expected drug response based on CellMiner database, *p* < 0.05 was considered valuable.

BMP1 expression significantly differed in the independent cohorts (Figure 9(c)). GSE35640, including 34 nonresponders and 22 responders, focused on MAGE-A3 immunotherapy in metastatic melanoma. In contrast, GSE78220 contained 13 nonresponders and 15 responders explored for MAPK-targeted therapy in melanoma. The IMvigor210 cohort containing 230 nonresponders and 68 responders was about urothelial cancer with atezolizumab intervention. The boxplots indicated that low BMP1 expression was notably related to patients with immunotherapeutic response in GSE78220, GSE35640, and IMvigor210. The expression level of BMP1 was positively related to drug response in patients treated with lenvatinib, idelalisib, pazopanib, rapamycin, everolimus, temsirolimus, abiraterone, zoledronate, bleomycin, and wortmannin. A negative relationship in the by-product of CUDC-305, palbociclib, oxaliplatin, amonafide, LDK-378, and dexrazoxane. The correlation between BMP1 and expected drug response is precisely displayed in Figure 10.

### 3.4. BMP1 CNV and Methylation Profile Analysis

**Figure 11:**
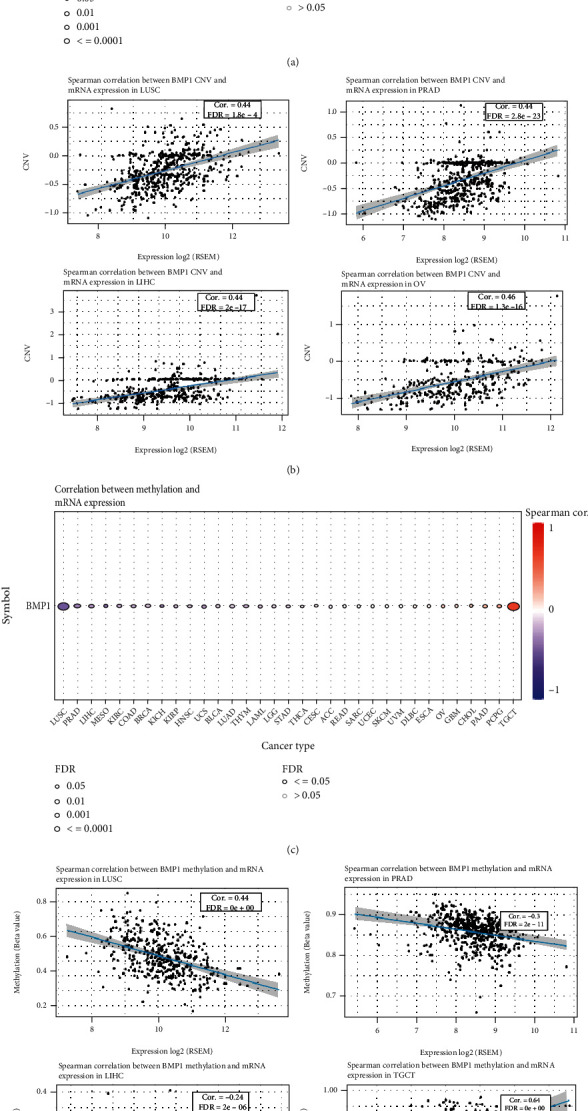
CNV and methylation profile analysis. (a) A connection between BMP1 mRNA expression and CNV. (b) The top four highest association scores between the mRNA expression and CNV. (c) A connection between BMP1 mRNA expression and methylation. (d) The top four highest association scores between mRNA expression and methylation.

The relationships between mRNA expression of BMP1 and CNV and methylation in various cancer types were explored based on the GSCA platform. In LUSC, PRAD, LIHC, and OV, a positive correlation between mRNA expression and BMP1 CNV were investigated. On the other hand, there was no statistically significant correlation in TGCT, ACC, PAAD, THYM, CHOL, LAML, LGG, HNSC, THCA, KIRC, KIRP, ESCA, and DLBC (Figure 11(a)). The plots on the right indicate the four highest correlation scores (Figure 11(b)).

The analysis also investigated an intimate connection between BMP1 mRNA expression and methylation in LUSC, PRAD, LIHC, and TGCT. In the exception of TGCT, we found the relevance of BMP1 methylation in LUSC, PRAD, and LIHC was substantial negative (Figure 11(c)). The plots on the right indicate the four highest correlation scores (Figure 11(d)).

## 4. Discussion

BMPs have been defined as potentially significant in tumor etiology. BMP1 was involved in the excitation of the TGF-*β* and BMP signaling pathways [23, 40]. Furthermore, overexpression of BMP1 was investigated in multifarious carcinomas, such as lung cancer, gastric cancer, and osteosarcoma [41–43]. Our analysis found an association between BMP1 and immune-related factors in pancancer. Comparing the tumor groups with normal groups, the expression of BMP1 was significantly related in 14 cancer types based on TCGA data. Furthermore, the connection of BMP1 expression with clinical parameters was explored. Significant age or neoplasm staging differences were identified in KIRC, LUAD, and THCA. It also indicated that upregulated BMP1 expression was connected with tumor tissues in KIRC, LUAD, and THCA, though there was no significant difference in LUAD. Significant differences in prognostic predictors (OS, DFS, and PFS) suggested BMP1 as a biomarker of poor prognosis in multiple carcinomas, for instance, ACC, LGG, MESO, and TGCT, which had not been researched before. The preceding conclusion demonstrated the potential value of BMP1 in pancancer prognosis, and a new hypothesis was proposed that therapeutic modulation of BMP1 activity in pancancer could translate into clinical benefits [29].

We identified BMP1 activity by comparing the transcriptional level in pancancer. It demonstrated that the transcription level might represent BMP1 activation in pancancer. The transcription level indicated BMP1 activation in MESO, UCS, SKCM, CHOL, OV, BRCA, THYM, PCPG, and KICH. However, there had inconsistency between BMP1 activity and expression in some types of cancers (DLBC and GBM). In addition, posttranscriptional protein level modification and protein metabolism might impact BMP1 expression.

The modification of m6A, one of the prevalent common RNA modifications, can influence the expression of partial cancer-related genes to impact tumor progression and metastasis [44, 45]. The previous article indicated that BMP1-processed IGFBP3 was significantly more effective in inhibiting Smad phosphorylation. Compared with intact IGFBP3, BMP1-cleaved IGFBP3 had an enhanced ability to inhibit FGF-induced proliferation [46]. We found that BMP1 was significantly related to IGFBP3, and in KIRP, BMP1 was positively associated with all the 20 m6A markers. We suggested that m6A modification may contribute to the promoting effect of BMP1 on KIRP. Furthermore, BMP1 impacted pancancer development and evolution through its association with IGFBP3.

Herein, we explored the association of BMP1 expression with immune cell infiltration. Furthermore, the intimate correlations between BMP1 and M0 macrophage content in DLBC, resting memory CD4 T cells in ACC, M2 macrophage infiltration, activated memory CD4 T cell, and naive B cell infiltration in TGCT are mentioned in this article. The data indicated that the variation of BMP1 expression affects the environment in macrophages. The tumor microenvironment (TME) landscapes we constructed implied that BMP1 may have a crucial effect on macrophage polarization. For immune inhibitors, immune stimulators, and MHC molecules, BMP1 showed a positive relationship with the majority of modulators, except ACC and TGCT. The phenomenon above hinted at the reflection of potential regulatory mechanisms in them. TGFB1 had the most significant positive association with BMP1 among multitudinous immune inhibitors in READ. TGFB1 (transforming growth factor-beta 1, TGF-*β*1) is a multifunctional signaling molecule that regulates immune function and inhibits the proliferation and activation of immune cells [47]. Previous evidence exhibited that a high expression of TGFB1 may have a worse prognosis by recruiting vasculature, reinforcing invasiveness and metastasis, and highlighting the significance of TGFB1 as a potential immunotherapeutic target of immune checkpoint blockade for rectal cancer [48, 49]. The underlying correlation between TGFB1 and BMP1 in rectal cancer must be analyzed and identified ulteriorly.

Furthermore, hippo-signaling pathways gathered in the high BMP1 expression groups of some tumors. The hippo-signaling pathway, a shining example of the clinical translation of basic immunology, contributes pivotal functions in carcinoma evolution and immunity, including innate and adaptive immune activation [50]. Our findings implied that high BMP1 expression might activate some tumors' innate immunity by regulating the hippo-signaling pathway. Additionally, we explored BMP1 expression in CNV and the methylation profile of human cancers to focus on the activity of regulatory factors and regulation of intracellular signaling, which is also considered momentous [51]. In some cancers (LUSC, PRAD, LIHC, and OV), a higher CNV meant a higher BMP1 expression. Furthermore, low methylation meant high BMP1 expression in some cancers (LUSC, PRAD, and LIHC). Consequently, the assessment of CNV and methylation might represent a reliable and complementary biomarker for predicting prognosis.

Finally, we explored the connection of the expression with an immunotherapeutic response based on the GEO database. Three cohorts showed significant differences in response and nonresponse (*p* < 0.05). The results indicated that a high expression of BMP1 contributes to poor immunotherapy response in melanoma and urothelial cancer, which had never been reported in previous papers. Previous research methods explored the underlying value of BMP1 in tumor immunotherapy and the connection of BMP expression with m6A modification and immune invasion. The findings in this article highlight the immunological effect of BMP1 in fractional carcinomas, but further research is required.

Our research still had many shortcomings. First, it was challenging to perform a correlation analysis of BMP1 at the protein level due to the lack of relevant data in public databases. Second, although three cohorts received and responded to anti-PD1 therapy, the small number of samples included in these three cohorts made it difficult to elucidate the actual immunotherapy response to BMP1. In the future, more relevant immunotherapy cohort studies should be conducted. Third, all studies were based on network analysis and machine computing, and we lacked corresponding experimental verification. We are currently developing relevant animal models for future BMP1 verification studies.

## 5. Conclusion

Our study determined that BMP1 expression in pancancer is associated with poor prognosis and highlights that BMP1 may be a potential clinical and therapeutic predictor in various cancers.

## Figures and Tables

**Table 1:** 33 types of human cancers employed in our research.

Abbreviation	Full name
ACC	Adrenocortical carcinoma
BLCA	Bladder urothelial carcinoma
BRCA	Breast invasive carcinoma
CESC	Cervical squamous cell carcinoma and endocervical adenocarcinoma
CHOL	Cholangiocarcinoma
COAD	Colon adenocarcinoma
DLBC	Diffuse large B cell lymphoma
ESCA	Esophageal carcinoma
GBM	Glioblastoma multiforme
HNSC	Head and neck squamous cell carcinoma
KICH	Kidney chromophobe
KIRC	Kidney renal clear cell carcinoma
KIRP	Kidney renal papillary cell carcinoma
LAML	Acute myeloid leukemia
LGG	Brain lower grade glioma
LIHC	Liver hepatocellular carcinoma
LUAD	Lung adenocarcinoma
LUSC	Lung squamous cell carcinoma
MESO	Mesothelioma
OV	Ovarian serous cystadenocarcinoma
PAAD	Pancreatic adenocarcinoma
PCPG	Pheochromocytoma and paraganglioma
PRAD	Prostate adenocarcinoma
READ	Rectum adenocarcinoma
SARC	Sarcoma
SKCM	Skin cutaneous melanoma
STAD	Stomach adenocarcinoma
TGCT	Testicular germ cell tumors
THCA	Thyroid carcinoma
THYM	Thymoma
UCEC	Uterine corpus endometrial carcinoma
UCS	Uterine carcinosarcoma
UVM	Uveal melanoma

## Data Availability

The data used to support the study's findings are either included in the article or can be found in online repositories.

## Abbreviations

BMP1: Bone morphogenetic protein 1
CNV: Copy number variations
CR: Complete response
GEO: Gene expression omnibus
GSCA: Gene set cancer analysis
GTEx: Genotype-tissue expression
MSI: Microsatellite instability
PR: Partial response
TCGA: The cancer genome atlas
TIMER: Tumor Immune estimation resource
TMB: Tumor mutational burden
UCSC: University of California Santa Cruz Xena explorer
UALCAN: University of Alabama at Birmingham Cancer.

## References

[1] Reddi A. H. (1996). BMP-1: resurrection as procollagen C-proteinase. *Science*.

[2] Rafi J. H., Jafar T., Pathan M. T. (2021). High expression of bone morphogenetic protein 1 (BMP1) is associated with a poor survival rate in human gastric cancer, a dataset approaches. *Genomics*.

[3] Kessler E., Takahara K., Biniaminov L., Brusel M., Greenspan D. S. (1996). Bone morphogenetic protein-1: the type I procollagen C-proteinase. *Science*.

[4] Ma H. Y., N'Diaye E. N., Caplazi P. (2022). BMP1 is not required for lung fibrosis in mice. *Scientific Reports*.

[5] Li S. W., Sieron A. L., Fertala A., Hojima Y., Arnold W. V., Prockop D. J. (1996). The C-proteinase that processes procollagens to fibrillar collagens is identical to the protein previously identified as bone morphogenic protein-1. *Proceedings of the National Academy of Sciences of the United States of America*.

[6] Pappano W. N., Steiglitz B. M., Scott I. C., Keene D. R., Greenspan D. S. (2003). Use of Bmp1/Tll1 doubly homozygous null mice and proteomics to identify and validate in vivo substrates of bone morphogenetic protein 1/tolloid-like metalloproteinases. *Molecular and Cellular Biology*.

[7] Hyytiainen M., Penttinen C., Keski-Oja J. (2004). Latent TGF-beta binding proteins: extracellular matrix association and roles in TGF-beta activation. *Critical Reviews in Clinical Laboratory Sciences*.

[8] Liu Y., Zhang Y., Tan Z. (2022). Lysyl oxidase promotes anaplastic thyroid carcinoma cell proliferation and metastasis mediated via BMP1. *Gland Surgery*.

[9] Slattery M. L., John E. M., Torres-Mejia G. (2013). Genetic variation in bone morphogenetic proteins and breast cancer risk in hispanic and non-hispanic white women: the breast cancer health disparities study. *International Journal of Cancer*.

[10] Kim M., Choe S. (2011). BMPs and their clinical potentials. *BMB Reports*.

[11] Xiao W., Wang X., Wang T., Xing J. (2020). Overexpression of BMP1 reflects poor prognosis in clear cell renal cell carcinoma. *Cancer Gene Therapy*.

[12] da Silva R., Uno M., Marie S. K., Oba-Shinjo S. M. (2015). LOX expression and functional analysis in astrocytomas and impact of IDH1 mutation. *PLoS One*.

[13] Singh A., Morris R. J. (2010). The Yin and Yang of bone morphogenetic proteins in cancer. *Cytokine & Growth Factor Reviews*.

[14] Zhu X., Luo X., Jiang S., Wang H. (2020). Bone morphogenetic protein 1 targeting COL1A1 and COL1A2 to regulate the epithelial-mesenchymal transition process of colon cancer SW620 cells. *Journal of Nanoscience and Nanotechnology*.

[15] Prieto T. G., Baldavira C. M., Machado-Rugolo J. (2021). Pulmonary neuroendocrine neoplasms overexpressing epithelial-mesenchymal transition mechanical barriers genes lack immune-suppressive response and present an increased risk of metastasis. *Frontiers in Oncology*.

[16] Cohn W. E. (1960). Pseudouridine, a carbon-carbon linked ribonucleoside in ribonucleic acids: isolation, structure, and chemical characteristics. *The Journal of Biological Chemistry*.

[17] Adams J. M., Cory S. (1975). Modified nucleosides and bizarre 5’-termini in mouse myeloma mRNA. *Nature*.

[18] Lee M., Kim B., Kim V. N. (2014). Emerging Roles of RNA Modification: m^6^A and U-Tail. *Cell*.

[19] Roundtree I. A., Evans M. E., Pan T., He C. (2017). Dynamic RNA modifications in gene expression regulation. *Cell*.

[20] Xu Y., Zhang M., Zhang Q. (2021). Role of main RNA methylation in hepatocellular carcinoma: N6-methyladenosine, 5-methylcytosine, and N1-methyladenosine. *Frontiers in Cell and Development Biology*.

[21] Frye M., Harada B. T., Behm M., He C. (2018). RNA modifications modulate gene expression during development. *Science*.

[22] Zaccara S., Ries R. J., Jaffrey S. R. (2019). Reading, writing and erasing mRNA methylation. *Nature Reviews. Molecular Cell Biology*.

[23] Bhattarai P. Y., Kim G., Poudel M., Lim S. C., Choi H. S. (2021). METTL3 induces PLX4032 resistance in melanoma by promoting m^6^A-dependent EGFR translation. *Cancer Letters*.

[24] Meyer K. D., Jaffrey S. R. (2017). Rethinking m6A readers, writers, and erasers. *Annual Review of Cell and Developmental Biology*.

[25] Wen J., Lv R., Ma H. (2018). Zc3h13 regulates nuclear RNA m^6^A methylation and mouse embryonic stem cell self-renewal. *Molecular Cell*.

[26] Su R., Dong L., Li Y. (2022). METTL16 exerts an m^6^A-independent function to facilitate translation and tumorigenesis. *Nature Cell Biology*.

[27] Gu Z., Du Y., Zhao X., Wang C. (2022). Diagnostic, therapeutic, and prognostic value of the m6A writer complex in hepatocellular carcinoma. *Frontiers in Cell and Development Biology*.

[28] Shen W. B., Ni J., Yao R. (2022). Maternal obesity increases DNA methylation and decreases RNA methylation in the human placenta. *Reproductive Toxicology*.

[29] Mo Z., Li P., Cao Z., Zhang S. (2021). A comprehensive pan-cancer analysis of 33 human cancers reveals the immunotherapeutic value of aryl hydrocarbon receptor. *Frontiers in Immunology*.

[30] Adib E., Nassar A. H., Akl E. W. (2021). CDKN2Aalterations and response to immunotherapy in solid tumors. *Clinical Cancer Research*.

[31] Li T., Fu J., Zeng Z. (2020). TIMER2.0 for analysis of tumor-infiltrating immune cells. *Nucleic Acids Research*.

[32] Yoshihara K., Shahmoradgoli M., Martinez E. (2013). Inferring tumour purity and stromal and immune cell admixture from expression data. *Nature Communications*.

[33] Liu Y., Wu Y., Zhang P. (2021). CXCL12 and CD3E as indicators for tumor microenvironment modulation in bladder cancer and their correlations with immune infiltration and molecular subtypes. *Frontiers in Oncology*.

[34] Newman A. M., Liu C. L., Green M. R. (2015). Robust enumeration of cell subsets from tissue expression profiles. *Nature Methods*.

[35] Ru B., Wong C. N., Tong Y. (2019). TISIDB: an integrated repository portal for tumor-immune system interactions. *Bioinformatics*.

[36] Cristescu R., Mogg R., Ayers M. (2018). Pan-tumor genomic biomarkers for PD-1 checkpoint blockade-based immunotherapy. *Science*.

[37] Bonneville R., Krook M. A., Kautto E. A. (2017). Landscape of microsatellite instability across 39 cancer types. *Oncologia*.

[38] Lara P. N., Redman M. W., Kelly K. (2008). Disease control rate at 8 weeks predicts clinical benefit in advanced non-small-cell lung cancer: results from southwest oncology group randomized trials. *Journal of Clinical Oncology*.

[39] Liu C. J., Hu F. F., Xia M. X., Han L., Zhang Q., Guo A. Y. (2018). GSCALite: a web server for gene set cancer analysis. *Bioinformatics*.

[40] Grgurevic L., Macek B., Healy D. R. (2011). Circulating bone morphogenetic protein 1-3 isoform increases renal fibrosis. *Journal of the American Society of Nephrology*.

[41] Wu X., Liu T., Fang O., Leach L. J., Hu X., Luo Z. (2014). miR-194 suppresses metastasis of non-small cell lung cancer through regulating expression of BMP1 and p27^kip1^. *Oncogene*.

[42] Hsieh Y. Y., Tung S. Y., Pan H. Y. (2018). Upregulation of bone morphogenetic protein 1 is associated with poor prognosis of late-stage gastric cancer patients. *BMC Cancer*.

[43] Garimella R., Tadikonda P., Tawfik O., Gunewardena S., Rowe P., van Veldhuizen P. (2017). Vitamin D impacts the expression of Runx2 target genes and modulates inflammation, oxidative stress and membrane vesicle biogenesis gene networks in 143B osteosarcoma cells. *International Journal of Molecular Sciences*.

[44] Shen C., Xuan B., Yan T. (2020). m6A-dependent glycolysis enhances colorectal cancer progression. *Molecular Cancer*.

[45] Chen H., Gao S., Liu W. (2021). RNA *N*^6^-methyladenosine methyltransferase METTL3 facilitates colorectal cancer by activating the m^6^A-GLUT1-mTORC1 axis and is a therapeutic target. *Gastroenterology*.

[46] Kim B., Huang G., Ho W. B., Greenspan D. S. (2011). Bone Morphogenetic Protein-1 Processes Insulin-like Growth Factor-binding Protein 3∗. *The Journal of Biological Chemistry*.

[47] Letterio J. J., Roberts A. B. (1998). Regulation of immune responses by TGF-*β*. *Annual Review of Immunology*.

[48] Sparks R., Bigler J., Sibert J. G., Potter J. D., Yasui Y., Ulrich C. M. (2004). TGFbeta1 polymorphism (L10P) and risk of colorectal adenomatous and hyperplastic polyps. *International Journal of Epidemiology*.

[49] Pezzolo E., Modena Y., Corso B., Giusti P., Gusella M. (2015). Germ line polymorphisms as predictive markers for pre-surgical radiochemotherapy in locally advanced rectal cancer: a 5-year literature update and critical review. *European Journal of Clinical Pharmacology*.

[50] Zhang Y., Zhang H., Zhao B. (2018). Hippo signaling in the immune system. *Trends in Biochemical Sciences*.

[51] Berg V., Rusch M., Vartak N. (2015). miRs-138 and -424 control palmitoylation-dependent CD95-mediated cell death by targeting acyl protein thioesterases 1 and 2 in CLL. *Blood*.

